# LncRNA LINC00460 promotes the papillary thyroid cancer progression by regulating the LINC00460/miR-485-5p/Raf1 axis

**DOI:** 10.1186/s40659-019-0269-9

**Published:** 2019-12-23

**Authors:** Guangjun Li, Qingli Kong

**Affiliations:** 1 Department of Oncology, Yuncheng County Hospital of Traditional Chinese Medicine, Heze, 274700 Shandong China; 2 Department of Oncology, The Affiliated Hospital of Jining Medical University, No. 89, Gutun Road, Jining, 272029 Shandong China

**Keywords:** LINC00460, miR-485-5p, Raf1, Papillary thyroid cancer, Progression

## Abstract

**Background:**

Papillary thyroid cancer (PTC) is the most common malignancy of all thyroid cancers. LncRNA LINC00460 has been proved to play roles in the oncogenesis and progression of various tumors, including papillary thyroid cancer. However, the potential molecular mechanism of LINC00460 in PTC is poorly investigated.

**Results:**

LINC00460 was upregulated in PTC tissues and cells. Raf1 was upregulated in PTC tissues, but miR-485-5p was down-regulated. High LINC00460 expression was associated with poor prognosis. LINC00460 knockdown suppressed proliferation, migration, invation and EMT of PTC cells. Bioinformatics prediction revealed that LINC00460 had binding sites with miR-485-5p, which was validated by luciferase reporter assay. In addition, miR-485-5p was confirmed to directly target Raf1 3′-UTR. Moreover, LINC00460 promoted PTC progression by sponging miR-485-5p to elevate the expression of Raf1. Knockdown of LINC00460 restrained tumor growth in vivo.

**Conclusion:**

LINC00460 induced proliferation, migration, invation and EMT of PTC cells by regulating the LINC00460/miR-485-5p/Raf1 axis, which indicated that LINC00460 may be a potential biomarker and therapeutic target for PTC.

## Background

Thyroid cancers are divided into four types: papillary thyroid cancer (PTC), follicular thyroid cancer (FTC), medullary thyroid cancer (MTC) and anaplastic thyroid cancer (ATC) [1]. Papillary thyroid cancer (PTC) is the most common malignancy of all thyroid cancers, accounting for approximately 80% of thyroid cancers and occurring frequently in females [2].

Long noncoding RNAs (lncRNAs) are highly conserved molecules longer than 200 nucleotides in length [3]. LncRNAs can serve as biomarker and therapeutic target during tumorigenesis and progression [4]. In thyroid cancer, lncRNAs have been to be novel therapeutic targets, diagnostic and prognostic markers [5]. Many lncRNAs downregulation regulated cell proliferation, migration and invasion in thyroid cancer using loss-of-function assays [6, 7]. However, lncRNA was not only an oncogene, but also a tumor suppressor in PTC progression [8]. Long noncoding RNA 00460 (LINC00460) has been reported to have regulatory effects in various cancers, such as promoting tumor growth of gastric cancer [9] and promoting colorectal cancer cells metastasis [10]. Among them, LINC00460 knockdown inhibited cell proliferation and invasion by inhibiting Wnt/β-catenin signaling in gastric cancer [9]. LINC00460 expedited the metastasis of lung cancer cells [11]. Besides, LINC00460 facilitated the development of head and neck squamous cell carcinoma via acting as a sponge of miR-612 and increasing AKT2 expression [12]. In previous studies, high-throughput sequencing revealed that LINC00460 was upregulated in thyroid cancer [13]. However, the mechanism of LINC00460 in PTC is poorly studied.

MicroRNAs (miRNAs) are highly conserved short noncoding RNAs composed of 18–25 nucleotides. Large numbers of miRNAs have been identified to act as oncogenes or tumor suppressors by modulating different target mRNAs [14]. A previous study revealed that miR-485-5p upregulation could facilitate osteosarcoma cell proliferation, migration and invasion by decreasing the expression of CX3CL1 [15]. Additionally, miR-485-5p has also been studied in thyroid cancer, and the results show that miR-485-5p was lowly expressed in thyroid cancer and regulated the progression of thyroid cancer [16]. However, the connection between LINC00460 and miR-485-5p in the progression of PTC has not been elucidated.

Raf1 (serine/threonine kinase) plays a role in the RAS/RAF/MEK/ERK signaling pathway to regulate tumor progression [17]. Raf1 has been studied to act as a tumor promoter in many cancers, such as osteosarcoma [18], gastric cancer [19], non-small cell lung cancer [17]. Raf1 was an important factor in promoting the tumorigenesis and progression of cancer. However, the role of Raf1 in PTC is rarely investigated.

In this study, the expression levels of LINC00460 and Raf1 were increased, and miR-485-5p expression was decreased in PTC tissues or cells. Knockdown of LINC00460 suppressed proliferation, migration, and invasion of PTC cell. Overall, LINC00460 promoted the papillary thyroid cancer progression by regulating LINC00460/miR-485-5p/Raf1 axis, which might provide novel biomarkers for PTC treatment.

## Materials and methods

### Tissue samples

All PTC tissues and adjacent normal tissues were collected from 58 patients who underwent surgery at Yuncheng county hospital of traditional Chinese medicine. Pathological examination confirmed that all patients were diagnosed. Written informed consent was obtained from all patients, and this study was approved by the Ethics Committee of Yuncheng county hospital of traditional Chinese medicine. All tissue samples were immediately frozen in liquid nitrogen and stored at − 80 °C. Some of the clinical features of patients are listed in Table 1. Patients with PTC were staged using the eight edition, TNM classification of American Joint Committee on Cancer.

**Table 1:** Correlation between clinicopathological features and LINC00460 expression in 58 patients with PTC

Parameters	Total	LINC00460 expression		*P*-value
		High (n = 29)	Low (n = 29)	
Age				
< 60	27	13	14	0.685
≥ 60	31	16	15	
Gender				
Male	20	12	8	0.262
Female	38	17	21	
Tumor size (cm)				
< 1	26	9	17	0.018*
≥ 1	32	20	12	
TNM stage				
I–II	34	14	20	0.025*
III–IV	24	15	9	
Lymph node metastasis				
No	33	14	19	0.038*
Yes	25	15	10	

### Cell culture

The human normal thyroid epithelial cell line (Nthy-ori 3-1) and the PTC cell lines (FTC-133 and 8505C) were purchased from the European Collection of Authenticated Cell Cultures (ECACC, Porton Down, UK). The PTC cell line (TPC1) were obtained from TOKU-E (Tokyo, Japan). The PTC cell line (BCPAP) were purchased from the German Collection of Microorganisms and Cell Cultures (DSMZ, Braunschweig, Niedersachsen, GER). All cell lines were maintained in Dulbecco’s Modified Eagle Medium (DMEM; Invitrogen, Carlsbad, CA, USA) supplemented with 10% fetal bovine serum (FBS; PAN, Adenbach, Bavaria, Germany) and cultured in a humid environment with 5% CO_2_ at 37 °C.

### Plasmids and cell transfection

Small interfering RNA (siRNA) of LINC00460 (si-LINC00460#1 and si-LINC00460#2) and the negative control siRNA (si-NC), LINC00460 overexpression plasmid (LINC00460) and the control pcDNA (Vector) were purchased from RiboBio (Guangzhou, China). MiR-485-5p mimics and the negative control mimics (miR-NC), miR-485-5p inhibitor (anti-miR-485-5p) and the corresponding negative control (anti-miR-NC), pcDNA-Raf1 (Raf1) and the control pcDNA (Vector) were synthesized by Genelily BioTech (Shanghai, China). Cell transfection was performed by Lipofectamine 2000 reagent (Invitrogen) according to the manufacturer’s instructions.

### Quantitative real-time PCR

Total RNA was extracted from tissues and cells using Trizol reagent (Invitrogen) following the protocols of manufacturer. RNA was reverse-transcribed into cDNA using the High-Capacity cDNA Reverse Transcription Kits (Thermo Fisher Scientific, Rockford, IL, USA) or SYBR PrimeScript miRNA RT-PCR Kit (Takara, Dalian, China). The expression levels were detected using SYBR Green Mixture (Takara) or SYBR PrimeScript miRNA RT-PCR Kit (Takara). GAPDH or U6 was detected as internal control. Primers as follows: LINC00460 (forward, 5′-GTGGATGAGAACGAAGGTTACG-3′; reverse, 5′-CTTTCCCACGCTCAGTCTTT-3′), miR-590-3p (forward, 5′-CCAAGCTTCACCCATTCCTAACAGGAC-3′; reverse, 5′-CGGGATCCGTAGGTCAGTTACATGCATC-3′), Raf1 (forward, 5′-GGGAGCTTGGAAGACGATCAG-3′; reverse, 5′-ACACGGATAGTGTTGCTTGTC-3′), GAPDH (forward, 5′-GACTCCACTCACGGCAAATTCA-3′; reverse, 5′-TCGCTCCTGGAAGATGGTGAT-3′), U6 (forward, 5′-CTCGCTTCGGCAGCACATATACT-3′; reverse, 5′-CGCTTCACGAATTTGCGTGT-3′).

### CCK-8 assay

The transfected TPC1 and BCPAP cells were plated in 96-well plates (Corning, Corning, NY, USA) at a density of 3.0 × 10^3^ cells per well. Then, a final 10% concentration of the Cell Counting Kit-8 (CCK-8; Dojindo, Kumamoto, Japan) was added to each well after incubation for 0 h, 24 h, 48 h and 72 h. After 2 h incubation at 37 °C, the absorbance was measured at 450 nm by Microplate Reader (Bio-Rad, Hercules, CA, USA). Each sample was prepared in triplicate.

### Transwell assay

Transwell chambers were used to evaluate cell migration and invasion ability, but transwell chambers for cell invasion were coated with Matrigel (BD Biosciences, Franklin Lakes, NJ, USA). Firstly, the transfected cells (5 × 10^4^ cell/well) were suspended in serum-free medium and then seeded into the upper chamber of a 24-well transwell with 8 μm polycarbonate membrane filters (Corning). The lower chamber contained 10% fetal bovine serum (FBS) as chemoattractant. Subsequently, cells were incubated for 48 h at 37 °C, the cells adhering to the lower surface were fixed with methanol and stained with crystal violet. Then, the cells were counted under the microscope and at least selected three fields.

### Western blot assay

Total protein was lysed from cells by RIPA lysis buffer (Thermo Fisher Scientific) supplemented with protease inhibitor (Thermo Fisher Scientific). Protein concentration was measured using the BCA Protein Assay Kit (Pierce, Appleton, WI, USA). Subsequently, the proteins were separated by sodium dodecyl sulfate polyacrylamide gel electrophoresis (SDS-PAGE) and transferred to polyvinylidene fluoride (PVDF) membranes (Millipore, Billerica, MA, USA). The membranes were blocked by 5% skim milk (Nestlé, Shuangcheng, China) for 2 h at room temperature and incubated with primary antibodies, anti-Raf1 (1:2000; Abcam, Cambridge, UK), anti-MMP9 (1:2000; Abcam), anti-E-cadherin (1:2000; Abcam), anti-N-cadherin (1:2000; Abcam) or anti-Vimentin (1:2000; Abcam) overnight at 4 °C. Then, the membranes were washed twice in TBST and incubated with the corresponding horseradish peroxidase (HRP)-conjugated secondary antibody (1:4000; Abcam) for 2 h at room temperature. Finally, the protein bands were visualized by enhanced chemiluminescence system (Thermo Fisher Scientific) and the intensity of the interest proteins was determined by ImageJ software (National Institutes of Health, Bethesda, MD, USA). GAPDH was used as endogenous reference.

### Luciferase reporter assay

The full fragments of LINC00460 (LINC00460-WT) containing predicted miR-485-5p binding sites or the mutant LINC00460 (LINC00460-MUT) including point mutations of the binding sites was inserted into pGL3 luciferase reporter plasmids (Promega, Madison, WI, USA). The corresponding vectors were cotransfected with miR-485-5p mimics or miR-NC into TPC1 and BCPAP cells using Lipofectamine 2000 (Invitrogen). Similarly, wild-type or mutant Raf1 3′-UTR fragments containing the targeting sites of miR-485-5p was cloned into pGL3 plasmids, respectively. Then, the miR-485-5p mimics or miR-NC was cotransfected with Raf1-WT or Raf1-MUT vector into TPC1 and BCPAP cells. Luciferase activity was detected using Luciferase Reporter Assay System (Promega) at 48 h after transfection as the manufacturers required.

### Xenograft experiment

BALB/c nude mice (5-week-old) were randomly divided into two groups (6 mice in each group). Lentivirus containing LINC00460 short hairpin RNA (shRNA) (sh-LINC00460) or negative control (sh-NC) was constructed by Genelily BioTech. Lentivirus carrying sh-LINC00460 or sh-NC was stably transfected into TPC1 cells (2 × 10^6^) and subcutaneously injected into the right abdomen of mice. Tumor volume was measured every 5 days. Thirty days later, the mice were killed and the xenograft was removed and weighed. The xenograft assay was ratified by the Animal Research Committee of Yuncheng county hospital of traditional Chinese medicine.

### Statistical analysis

All date were shown as mean ± standard deviation (SD) and had three independent experiments. Graphpad Prism 7.0 software (GraphPad, San Diego, CA, USA) was used for statistical analysis. The differences were analyzed by Student’s *t*-test and one-way analysis of variance (one-way ANOVA). *P*-value < 0.01 was considered that the difference is extremely significant.

## Results

### LINC00460 was upregulated in PTC tissues and cells and related to poor prognosis

**Fig. 1:**
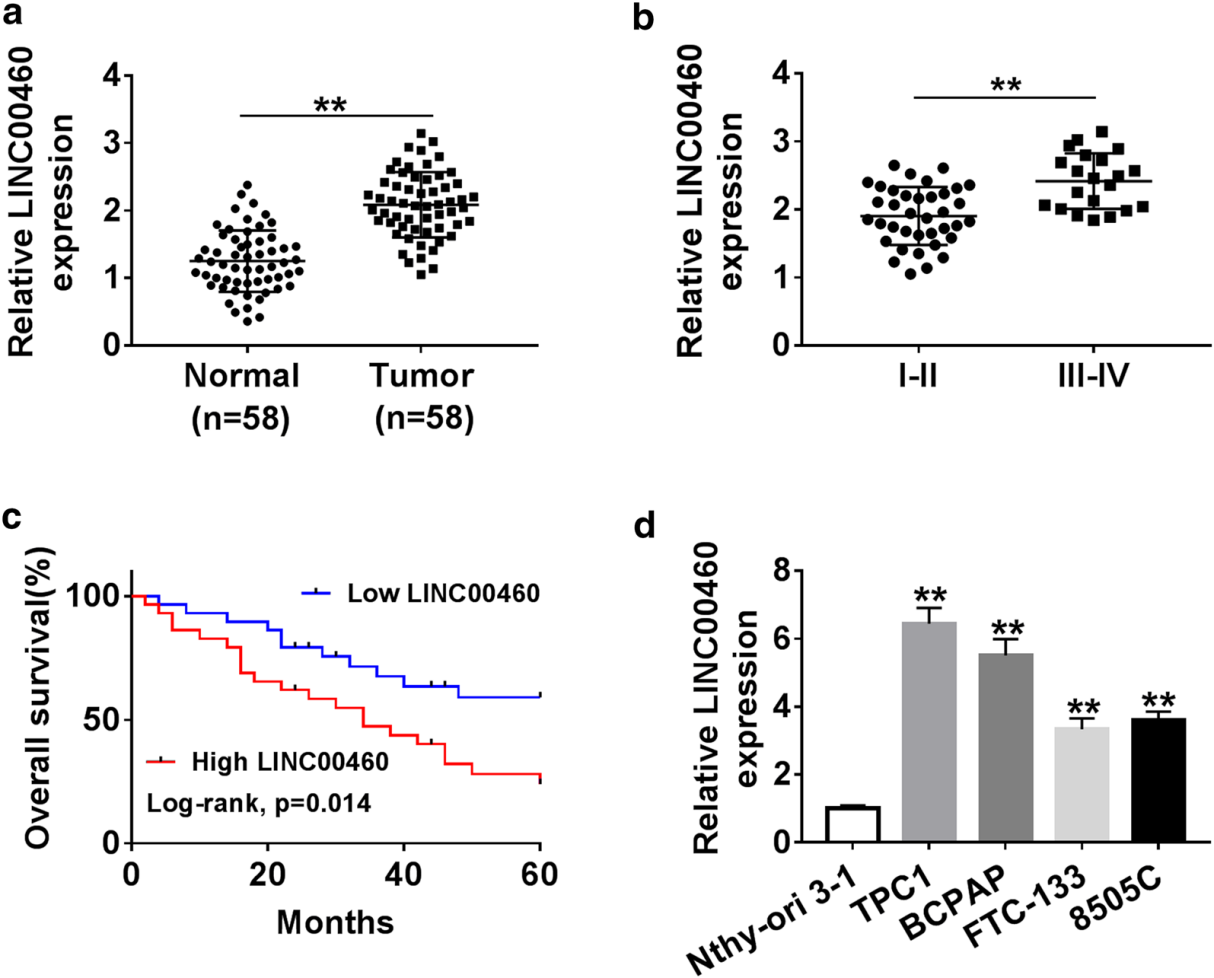
LINC00460 was upregulated in PTC tissues and cells and related to poor prognosis. **a** qRT-PCR assay was utilized to measure LINC00460 expression in PTC tissues and adjacent normal tissues. **b** The expression of LINC00460 was detected in patients with different TNM stage by qRT-PCR. **c** Kaplan–Meier survival analysis was carried out to analyze the correlation between LINC00460 expression and overall survival rate. **d** The LINC00460 expression was detected in normal thyroid epithelial cell line (Nthy-ori 3-1) and the PTC cell lines (TPC1, BCPAP, FTC-133 and 8505C). **P < 0.01

Firstly, the expression of LINC00460 in 58 PTC tissues and adjacent normal thyroid tissues was detected by qRT-PCR. The results revealed that LINC00460 expression was significantly increased in PTC tissues compared with the adjacent non-tumor tissues (Fig. 1a). We also investigated some clinical pathological status of patients with different expression levels of LINC00460 and the data showed that LINC00460 expression was significantly higher in patients with advanced tumor node metastasis (TNM) stage (Table 1 and Fig. 1b). Furthermore, LINC00460 expression was not associated with patient age or gender, but was associated with tumor size and lymph node metastasis (LNM) (Table 1). In addition, we divided PTC samples into two groups with low or high expression of LINC00460 to evaluate the correlation between LINC00460 expression and the overall survival rate of PTC patients using Kaplan–Meier survival analysis, the results showed that high LINC00460 expression obviously shortened the patient’s survival time (Fig. 1c). Finally, the results of qRT-PCR revealed that LINC00460 expression was dramatically increased in the four PTC cell lines (TPC1, BCPAP, FTC-133 and 8505C) compared with Nthy-ori 3-1 (Fig. 1d). These results implied that high LINC00460 expression was associated with poor prognosis.

### LINC00460 knockdown inhibited proliferation, migration and invasion in PTC cells

**Fig. 2:**
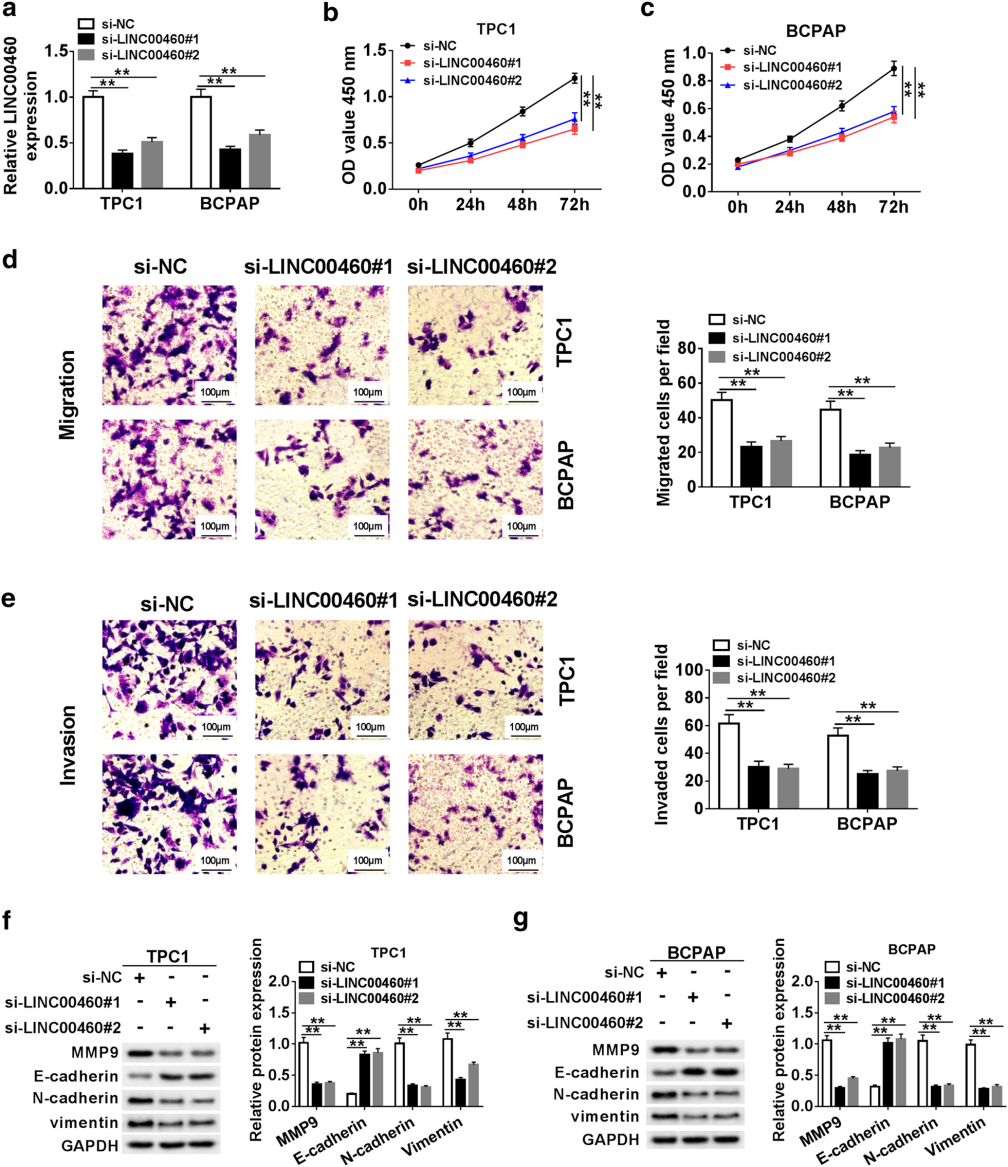
LINC00460 knockdown inhibited proliferation, migration and invasion in PTC cells. **a**–**g** TPC1 and BCPAP cells were transfected with si-NC, si-LINC00460#1 or si-LINC00460#2. **a** The expression of LINC00460 was detected by qRT-PCR in transfected cells. **b**, **c** Cell proliferation was evaluated using CCT-8 assay at 0 h, 24 h, 48 h and 72 h after transfection. **d**, **e** The migrated and invaded cells were measured by transwell assay. **f**, **g** The levels of EMT-related protein (MMP9, E-cadherin, N-Cadherin and Vimentin) were examined by western blot. **P < 0.01

To investigate the function of LINC00460 on proliferation, migration and invasion of PTC cells, TPC1 and BCPAP cells were transfected with si-LINC00460#1 or si-LINC00460#2 to suppress the expression of LINC00460. The results of qRT-PCR revealed that LINC00460 knockdown significantly down-regulated LINC00460 expression compared to the negative control and si-LINC00460#1 had higher knockdown efficiency (Fig. 2a). CCK-8 assay and transwell assay demonstrated that LINC00460 knockdown could remarkably suppressed cell proliferation (Fig. 2b, c), migration and invation (Fig. 2d, e) in TPC1 and BCPAP cells compared with cells transfected with si-NC. Western blot assay showed that knockdown of LINC00460 significantly increased the protein level of E-cadherin and dramatically decreased the protein levels of MMP9, N-cadherin and vimentin in TPC1 and BCPAP cells (Fig. 2f, g). These data suggested that LINC00460 knockdown suppressed proliferation, migration and invation of PTC cells.

### LINC00460 regulated Raf1 by sponging miR-485-5p in PTC cells

**Fig. 3:**
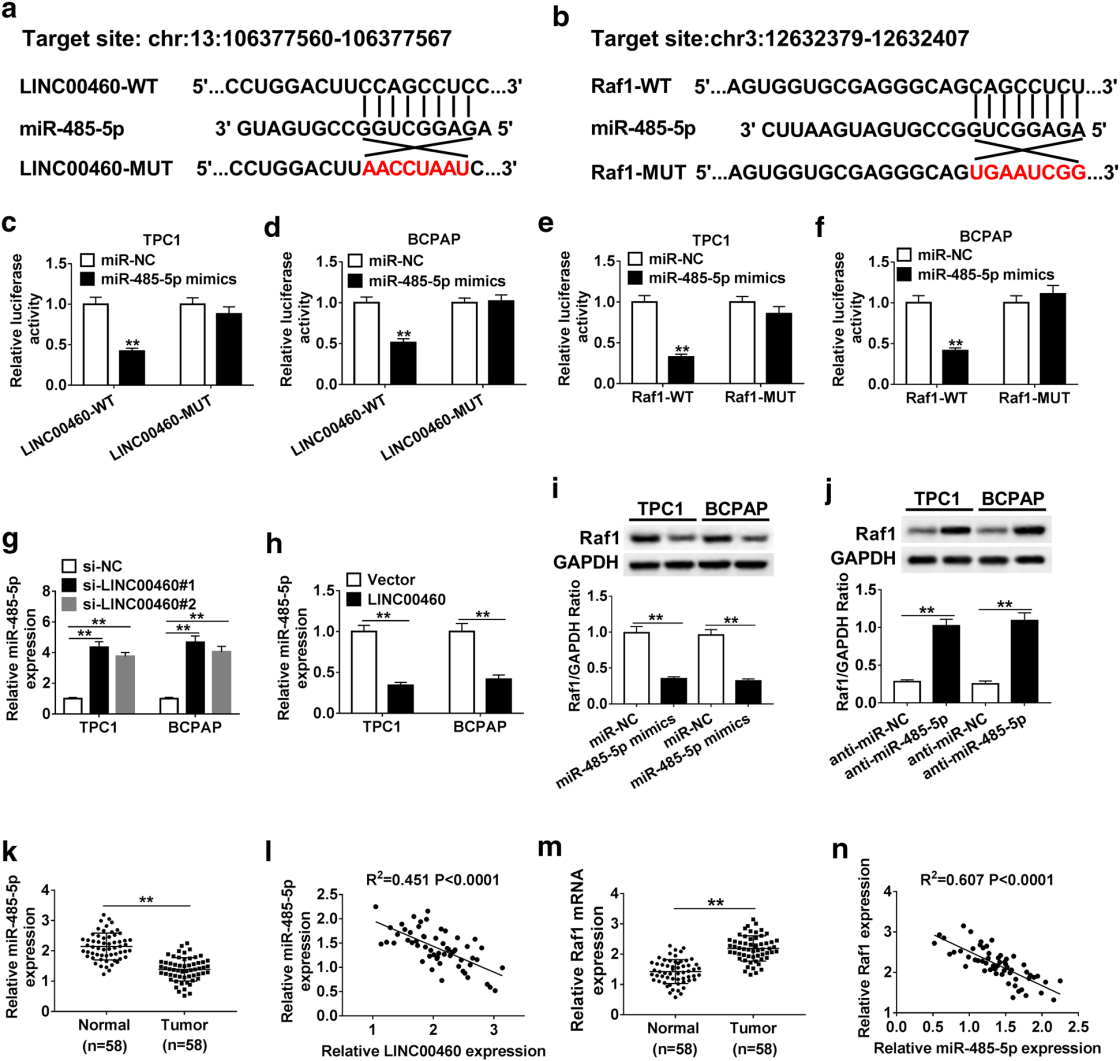
LINC00460 regulated Raf1 by sponging miR-485-5p in PTC cells. **a**, **b** The predicted binding sites of LINC00460 and miR-485-5p, miR-485-5p and Raf1 3′-UTR. **c**, **d** Luciferase activity was examined in TPC1 and BCPAP cells cotransfected with LINC00460-WT or LINC00460-MUT and miR-485-5p mimics or miR-NC. **e**, **f** TPC1 and BCPAP cells were cotransfected with Raf1-WT or Raf1-MUT and miR-485-5p mimics or miR-NC, and luciferase activity was detected. **g**, **h** TPC1 and BCPAP cells were transfected with si-NC, si-LINC00460#1, si-LINC00460#2, pcDNA-LINC00460 (LINC00460) and pcDNA (Vector), respectively. The expression of miR-485-5p were detected by qRT-PCR after transfection. **i**, **j** The protein level of Raf1 was detected in TPC1 and BCPAP cells transfected with miR-NC, miR-485-5p mimics, anti-miR-NC and anti-miR-485-5p, respectively. **k** The expression of miR-485-5p in normal tissues and PTC tissues were examined by qRT-PCR. **l** The correlation between LINC00460 and miR-485-5p. **m** Raf1 expression in normal tissues and PTC tissues was detected by qRT-PCR. **n** The correlation between miR-485-5p and Raf1 in PTC tissues. **P < 0.01

LINC00460 was predicted to have potential binding sites with miR-485-5p by bioinformatic software LncBase Predicted v.2 (Fig. 3a). StarBase v2.0 software predicted miR-485-5p binding to Raf1 (Fig. 3b). Then luciferase reporter assay was performed to verify these targeting relationships. The results revealed that mature miR-485-5p strikingly inhibited the luciferase activity of TPC1 and BCPAP cells transfected with LINC00460-WT or Raf1-WT, whereas miR-485-5p mimics could not modulate the luciferase activity when the binding sites were mutated (Fig. 3c–f). Next, the expression of miR-485-5p was detected in TPC1 and BCPAP cells transfected with si-NC, si-LINC00460#1, si-LINC00460#2, pcDNA-LINC00460 (LINC00460) and pcDNA (Vector), respectively. The results confirmed that LINC00460 knockdown strikingly upregulated miR-485-5p expression, while overexpression of LINC00460 significantly decreased miR-485-5p expression in TPC1 and BCPAP cells (Fig. 3g, h). Moreover, results of western blot revealed that miR-485-5p mimics markedly repressed the protein level of Raf1, and miR-485-5p inhibitor specially induced the protein level of Raf1 in TPC1 and BCPAP cells compared to negative control (Fig. 3i, j). Moreover, miR-485-5p expression was obviously decreased in PTC tissues compared with the normal tissues (Fig. 3k), and the expression between LINC00460 and miR-485-5p was negatively correlated in PTC tissues (Fig. 3l). In addition, the expression of Raf1 was significantly increased in PTC tissues compared with normal tissues (Fig. 3m), and Raf1 expression was negatively correlated with miR-485-5p expression in PTC tissues (Fig. 3n). These data suggested that LINC00460 regulated Raf1 expression by sponging miR-485-5p in PTC.

### Inhibition of miR-485-5p reversed the effects of LINC00460 knockdown in PTC cells

**Fig. 4:**
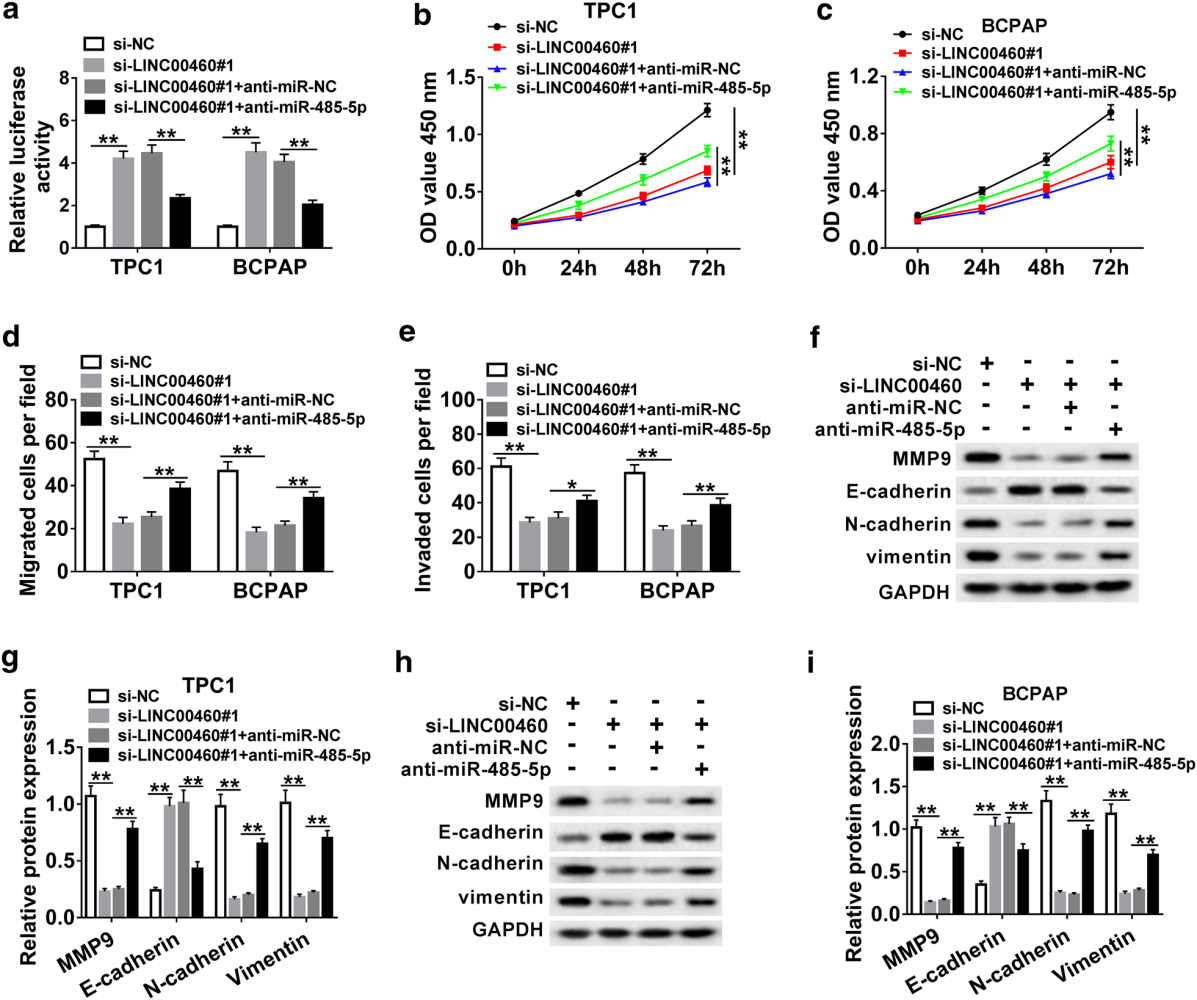
Inhibition of miR-485-5p reversed the effects of LINC00460 knockdown in PTC cells. **a**–**i** TPC1 and BCPAP cells were transfected with si-NC, si-LINC00460#1, si-LINC00460#1 + anti-miR-NC and si-LINC00460#1 + anti-miR-485-5p, respectively. **a** The expression of miR-485-5p was detected by qRT-PCR. **b**, **c** Cell proliferation was detected by CCK-8 assay at 0 h, 24 h, 48 h and 72 h after transfection. **d** The migrated cells were measured by transwell assay. **e** The invaded capacity was evaluated by transwell assay after transfection. **f**–**i** EMT-related proteins (MMP9, E-cadherin, N-Cadherin and Vimentin) expression was detected by western blot. **P < 0.01

To further investigated the effects of LINC00460 and miR-485-5p on proliferation, migration and invasion of PTC cells, TPC1 and BCPAP cells were transfected with si-NC, si-LINC00460#1, si-LINC00460#1 + anti-miR-NC and si-LINC00460#1 + anti-miR-485-5p, respectively. The results showed that inhibition of LINC00460 significantly upregulated miR-485-5p expression, while the expression of miR-485-5p was markedly decreased after knockdown of LINC00460 and miR-485-5p (Fig. 4a). Moreover, knockdown of LINC00460 remarkedly suppressed cell proliferation (Fig. 4b, c), migration (Fig. 4d) and invasion (Fig. 4e) of TPC1 and BCPAP cells. In addition, the protein level of E-cadherin distinctly increased and the protein levels of MMP9, N-cadherin and Vimentin dramatically reduced in TPC1 and BCPAP cells transfected with si-LINC00460#1 compared to the negative control (Fig. 4f–i). However, these effects caused by LINC00460 silencing could be abrogated by the si-LINC00460#1 + anti-miR-485-5p group, which suggested that knockdown of miR-485-5p reversed the inhibition of LINC00460 knockdown on PTC progression.

### MiR-485-5p regulated PTC cell progression through modulating Raf1

**Fig. 5:**
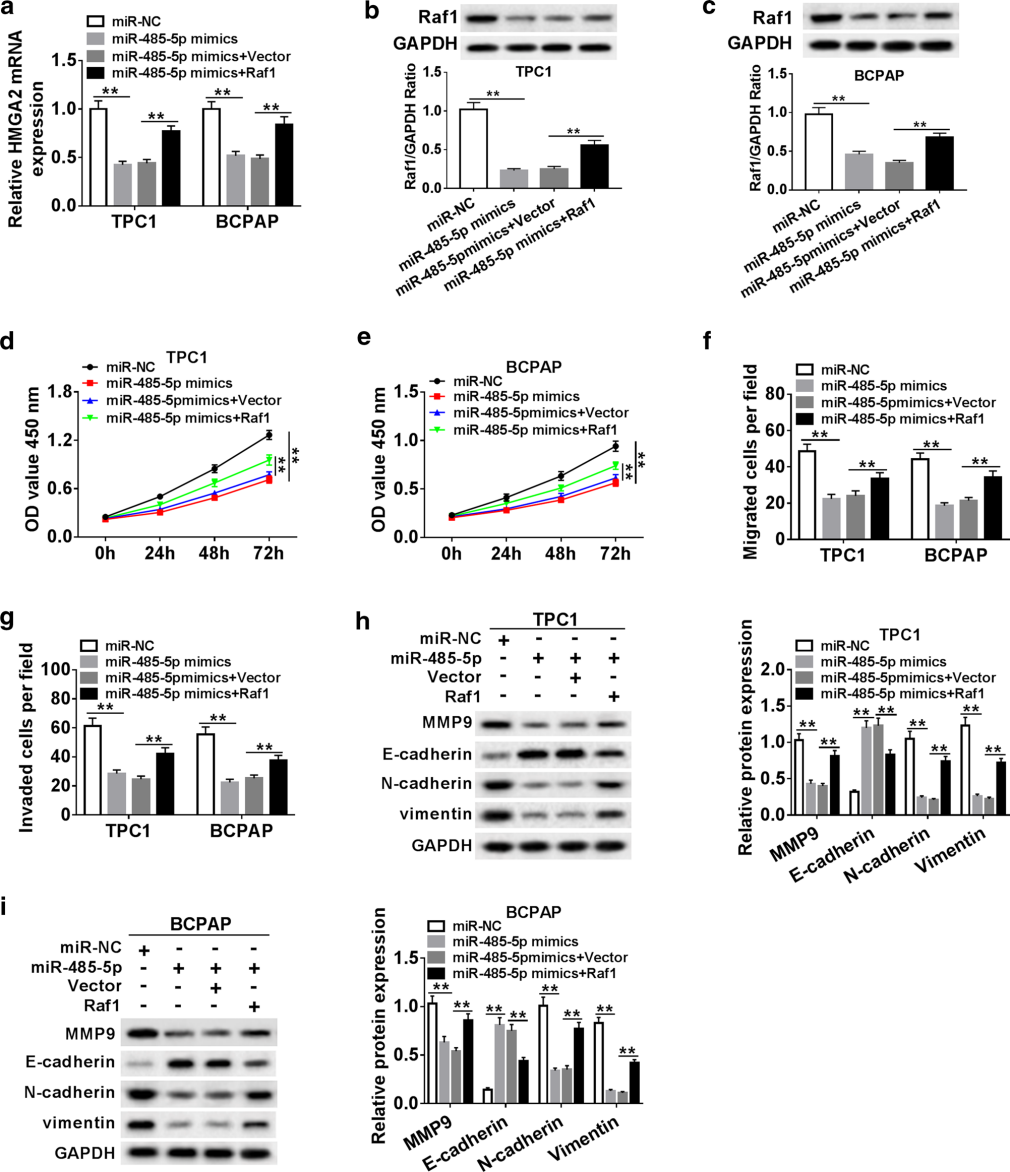
MiR-485-5p regulated PTC cell progression through modulating Raf1. **a**–**i** TPC1 and BCPAP cells were transfected with miR-NC, miR-485-5p mimics, miR-485-5p mimics + Vector, and miR-485-5p mimics + Raf1, respectively. **a** The expression of Raf1 was detected by qRT-PCR. **b**, **c** The protein level of Raf1 was detected by western blot in TPC1 and BCPAP cells, respectively. **d**, **e** Cell proliferation was detected by CCK-8 assay at 0 h, 24 h, 48 h and 72 h after transfection. **f** The migrated cells were measured by transwell assay. **g** The invaded capacity was evaluated by transwell assay after transfection. **h**, **i** EMT-related proteins (MMP9, E-cadherin, N-Cadherin and Vimentin) expression was detected by western blot. **P < 0.01

To further determine the effects of miR-485-5p and Raf1 in PTC progression, TPC1 and BCPAP cells were transfected with miR-NC, miR-485-5p mimics, miR-485-5p mimics + Vector, and miR-485-5p mimics + Raf1, respectively. The results showed that overexpression of miR-485-5p significantly down-regulated Raf1 expression, whereas the expression of Raf1 was significantly increased when overexpressing miR-485-5p and Raf1 (Fig. 5a). Western blot revealed that the protein level of Raf1 was strikingly down-regulated in TPC1 and BCPAP cells transfected with miR-485-5p mimics (Fig. 5b, c). Moreover, CCK-8 and transwell assays showed that miR-485-5p overexpression obviously suppressed proliferation (Fig. 5d, e), migration (Fig. 5f) and invasion (Fig. 5g) of TPC1 and BCPAP cells, whereas Raf1 overexpression in the meantime reversed the effects caused by overexpression of miR-485-5p. The protein level of E-cadherin significantly increased and the protein levels of MMP9, N-cadherin and Vimentin markedly decreased in TPC1 and BCPAP cells transfected with miR-485-5p mimic, which were abrogated by Raf1 overexpression (Fig. 5h, i). These results indicated that miR-485-5p regulated PTC cell proliferation, migration and invasion by modulating Raf1.

### LINC00460 regulated Raf1 expression through miR-485-5p in PTC cells

**Fig. 6:**
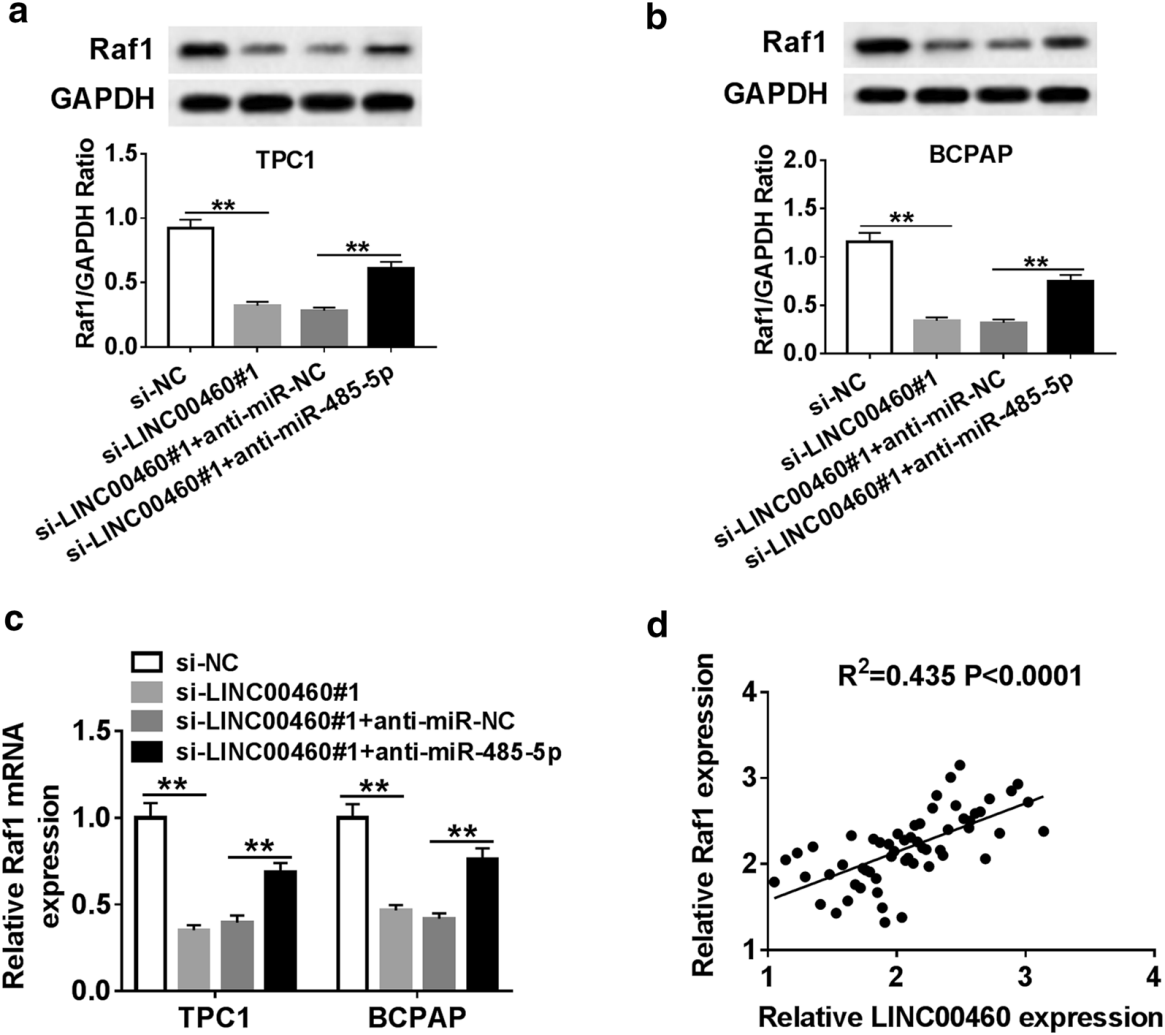
LINC00460 regulated Raf1 expression through miR-485-5p in PTC cells. **a**–**c** TPC1 and BCPAP cells were transfected with si-NC, si-LINC00460#1, si-LINC00460#1 + anti-miR-NC, si-LINC00460#1 + anti-miR-485-5p, respectively. **a**, **b** The protein level of Raf1 were measured by western blot. **c** The mRNA expression of Raf1 was detected by qRT-PCR. (D) The correlation between Raf1 and Raf1 was investigated. **P < 0.01

To study the relationship between LINC00460, miR-485-5p and Raf1, TPC1 and BCPAP cells were transfected with si-NC, si-LINC00460#1, si-LINC00460#1 + anti-miR-NC, si-LINC00460#1 + anti-miR-485-5p, respectively. The results indicated that LINC00460 knockdown significantly inhibited the protein level of Raf1 in TPC1 and BCPAP cells, while the protein level in the si-LINC00460#1 + anti-miR-485-5p group returned to normal (Fig. 6a, b). The mRNA expression of Raf1 was dramatically down-regulated in TPC1 and BCPAP cells transfected with si-LINC00460#1, whereas the effect was reversed in cells transfected with si-LINC00460#1 + anti-miR-485-5p (Fig. 6c). In addition, the correlation between LINC00460 and Raf1 was positively correlated (Fig. 6d). These results indicated that LINC00460 modulated Raf1 expression by regulating miR-485-5p in PTC cells.

### Depletion of LINC00460 inhibited tumor growth in vivo

**Fig. 7:**
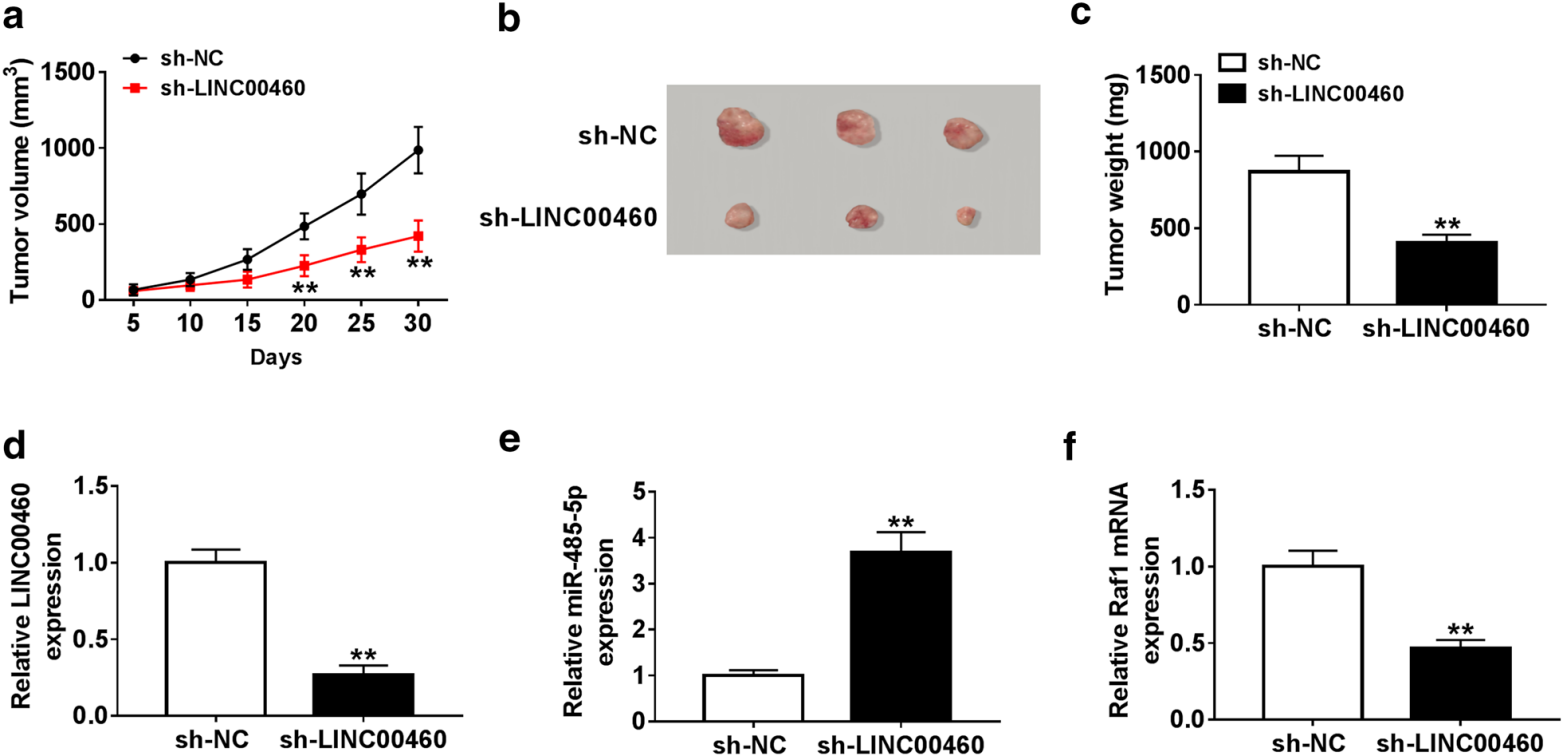
Depletion of LINC00460 inhibited tumor growth in vivo. TPC1 cells transfected with sh-LINC00460 or sh-NC were subcutaneously injected into nude mice. **a** Tumor volume was measured every 5 days. **b**, **c** The tumors were photographed and weight after mice were killed. **d**–**f** The levels of LINC00460, miR-485-5p and Raf1 were detected by qRT-PCR. **P < 0.01

To investigate the effect of LINC00460 on tumorigenesis, we established a xenograft mouse model. The results revealed that tumor volume and weight were evidently decreased in the sh-LINC00460 group relative to the sh-NC group (Fig. 7a–c). In addition, LINC00460 silencing prominently reduced the levels of LINC00460 and Raf1 and drastically increased the level of miR-485-5p (Fig. 7d–f). These data indicated that knockdown of LINC00460 impeded tumor growth in vivo.

## Discussion

In recent years, several lncRNAs associated with the progression of papillary thyroid cancer have been identified. Liang et al. [7] reported that MCM3AP-AS1 promoted cell proliferation, migration, and invasion by regulating the MCM3AP-AS1/miR-211-5p/SPARC axis in papillary thyroid cancer. Yuan et al. [20] found that knockdown of HOTTIP supressed the proliferation, migration and invasion of PTC cells through regulating miR-637, which revealed that HOTTIP is a therapeutic target for PTC. Feng et al. [21] confirmed that LINC00460 was an oncogene for PTC and promoted the progression of papillary thyroid cancer by regulating miR-613, suggesting that LINC00460 may be a potential therapeutic target for papillary thyroid cancer. However, the role of LINC00460 in PTC has been rarely studied. In this study, we found that LINC00460 was significantly upregulated in PTC tissues and cell lines, and LINC00460 knockdown inhibited proliferation, migration and invasion through regulating the miR-485-5p/Raf1 axis.

LncRNAs have been reported to suppress the targeting of mRNAs by miRNAs as competing endogenous RNAs (ceRNAs) [22]. This study revealed that LINC00460 could directly bind with miR-485-5p by bioinformatics analysis. Subsequently, LINC00460 sponging miR-485-5p was verified by luciferase reporter assay. MiR-485-5p has been widely reported as a tumor inhibitor in multiple cancers, such as colorectal cancer [23], osteosarcoma [15], breast cancer [24] et al. In glioma, miR-485-5p was dramatically down-regulated and suppressed cell proliferation, and promoted glioma cells cycle arrest in G1 by binding to paired box 3 (PAX3) [25]. Wang et al. revealed that long noncoding RNA DSCR8 acted as a competing endogenous RNA (ceRNA) via sponge miR-485-5p and activated Wnt/β-catenin signal pathway by regulating DSCR8/miR-485-5p/Frizzled-7 (FZD7) axis in hepatocellular carcinoma [26]. Further investigation by Zhang et al. [16] showed that miR-485-5p was competitively combined by FOXD2-AS1 to increase KLK7 expression and inhibited PTC progression. Our study further confirmed that miR-485-5p was significantly down-regulated in PTC tissues and cells. In this study, we showed that miR-485-5p could be regulated by LINC00460 and abolished the effect of LINC00460 in PTC, which suggested that miR-485-5p is a tumor suppressor in PTC.

Moreover, this research proved that LINC00460 could competitively binded with miR-485-5p to regulate Raf1 expression. Previous studies have indicated that Raf1 was a target of miR-431-5p and functioned as a promoter in angiogenesis and progression of lung cancer [27]. In thyroid cancer, Raf1, a direct target of miR-195, was significantly upregulated in thyroid carcinomas compared to benign tumors and miR-195 overexpression dramatically decreased the protein level of Raf1 and inhibited cell proliferation in thyroid cancer [28]. The present study showed that Raf1 was a direct target of miR-485-5p to function as a tumor promoter.

## Conclusion

This study suggested that LINC00460 was upregulated in papillary thyroid cancer tissues and cells and knockdown of LINC00460 inhibited proliferation, migration, invation and epithelial-to-mesenchymal transition (EMT) of PTC cells. LINC00460 regulated Raf1 by sponging miR-485-5p and miR-485-5p silencing reversed the effects of LINC00460 knockdown in PTC cells. In a word, LINC00460 promoted the papillary thyroid cancer progression by regulating the LINC00460/miR-485-5p/Raf1 axis, which provides a theoretical basis on PTC markers and potential therapeutic targets.

## Data Availability

All data generated and analysed during this study are included in this published article are available on request.

## Abbreviations

PTC: papillary thyroid cancer
FTC: follicular thyroid cancer
MTC: medullary thyroid cancer
ATC: anaplastic thyroid cancer
LINC00460: long noncoding RNA 00460
SDS-PAGE: sodium dodecyl sulfate polyacrylamide gel electrophoresis
FBS: fetal bovine serum
HRP: horseradish peroxidase
